# Effects of osteoporosis on the biomechanics of various supplemental fixations co-applied with oblique lumbar interbody fusion (OLIF): a finite element analysis

**DOI:** 10.1186/s12891-022-05645-7

**Published:** 2022-08-19

**Authors:** Zi-Xuan Liu, Zi-Wei Gao, Chao Chen, Zi-Yang Liu, Xin-Yi Cai, Ya-Nan Ren, Xun Sun, Xin-Long Ma, Cheng-Fei Du, Qiang Yang

**Affiliations:** 1 Tianjin Key Laboratory for Advanced Mechatronic System Design and Intelligent Control, School of Mechanical Engineering, Tianjin University of Technology, 391 Binshui West Road, Xiqing District, Tianjin, 300384 China; 2 Department of Spine Surgery, Tianjin Hospital, Tianjin University, 406 Jiefang South Road, Tianjin, 300211 China; 3 Tianjin Medical University, Tianjin, 300070 China

**Keywords:** Finite element analysis, Osteoporosis, Oblique lumbar interbody fusion, Various supplemental fixations, Biomechanical

## Abstract

**Background:**

Oblique lumbar interbody fusion (OLIF) is an important surgical modality for the treatment of degenerative lumbar spine disease. Various supplemental fixations can be co-applied with OLIF, increasing OLIF stability and reducing complications. However, it is unclear whether osteoporosis affects the success of supplemental fixations; therefore, this study analyzed the effects of osteoporosis on various supplemental fixations co-applied with OLIF.

**Methods:**

We developed and validated an L3-S1 finite element (FE) model; we assigned different material properties to each component and established models of the osteoporotic and normal bone lumbar spine. We explored the outcomes of OLIF combined with each of five supplemental fixations: standalone OLIF; OLIF with lateral plate fixation (OLIF + LPF); OLIF with translaminar facet joint fixation and unilateral pedicle screw fixation (OLIF + TFJF + UPSF); OLIF with unilateral pedicle screw fixation (OLIF + UPSF); and OLIF with bilateral pedicle screw fixation (OLIF + BPSF). Under the various working conditions, we calculated the ranges of motion (ROMs) of the normal bone and osteoporosis models, the maximum Mises stresses of the fixation instruments (MMSFIs), and the average Mises stresses on cancellous bone (AMSCBs).

**Results:**

Compared with the normal bone OLIF model, no demonstrable change in any segmental ROM was apparent. The MMSFIs increased in all five osteoporotic OLIF models. In the OLIF + TFJF + UPSF model, the MMSFIs increased sharply in forward flexion and extension. The stress changes of the OLIF + UPSF, OLIF + BPSF, and OLIF + TFJF + UPSF models were similar; all stresses trended upward. The AMSCBs decreased in all five osteoporotic OLIF models during flexion, extension, lateral bending, and axial rotation. The average stress change of cancellous bone was most obvious under extension. The AMSCBs of the five OLIF models decreased by 14%, 23.44%, 21.97%, 40.56%, and 22.44% respectively.

**Conclusions:**

For some supplemental fixations, the AMSCBs were all reduced and the MMSFIs were all increased in the osteoporotic model, compared with the OLIF model of normal bone. Therefore, the biomechanical performance of an osteoporotic model may be inferior to the biomechanical performance of a normal model for the same fixation method; in some instances, it may increase the risks of fracture and internal fixation failure.

## Background

Oblique lumbar interbody fusion (OLIF) is an important treatment for disc degeneration and has become very popular among both physicians and patients. OLIF was first proposed by Mayer in 1997 [1]. Later, Silvestre et al. [2] improved the procedure by formally delivering OLIF through a channel between the peritoneum and the psoas major muscle, thus causing minimal invasion of the intervertebral disc space. Compared with other lumbar interbody fusions, the advantages of OLIF include less blood loss and shorter surgery; patients recover quickly and can be rapidly discharged [3, 4]. During OLIF, the lumbar spine is accessed through a window between the anterior blood vessel and the psoas muscle. Thus, OLIF does not damage the posterior structures and can effectively treat intervertebral disc degeneration by retaining more bone mass; this is important in patients with osteoporosis.

Osteoporotic bone is defined as “a type of bone characterized by low bone mass and microstructural degradation caused by bone tissue diseases, the results of which are bone fragility and increased risk of fractures” [5, 6]. In clinical practice, osteoporotic patients with degenerative lumbar spine disease often require OLIF to increase lumbar stability and reduce the risks of lumbar fracture and failure; the optimal supplemental fixation required when performing OLIF in an osteoporotic patient remains unclear. An appropriate supplemental fixation choice is particularly important in such patients. Screw loosening and extraction have been reported during follow-up of osteoporotic patients who had undergone fusion surgery for internal fixation. Fractures have also been reported; these may require surgical intervention that increases the financial strain for osteoporotic patients [7]. Few biomechanical studies have appeared concerning the effects of osteoporosis on various supplemental fixations co-applied with OLIF. These focused on intraoperative observation, postoperative follow-up, and summaries of prior works [2, 3, 8, 9]. Biomechanics clearly has important effects on the physiology, pathology, and surgical repair outcomes of the lumbar spine [10–12]. It is appropriate for clinicians to study the combinations of OLIF with various supplemental fixations for patients with osteoporosis.

The finite element (FE) method is widely used to explore mechanical issues, such as the biomechanics of the lumbar spine [13–17]. Traditional mechanical testing requires clinical specimens or cadavers; however, sourcing of in vitro specimens is difficult and time-consuming. Furthermore, soft tissues such as ligaments and muscles tend to fatigue after death; thus, the results may not consider the important interactions between bone and other tissues. However, FEs can be simulated in the absence of clinical specimens or cadavers, thus partially compensating for the shortage of in vitro data [18–20]. The FE method can be used to simulate the various supplemental fixations, aiding physicians in the development of appropriate treatment plans and selection of optimal surgeries [21]. Thus, considering the advantages of the FE method in the context of a biomechanical problem, we used this method to explore the effects of osteoporosis on the biomechanics of various supplemental fixations co-applied with OLIF.

We built a complete three-dimensional FE model of the L3-S1 lumbar spine and sacrum, then simulated five supplemental fixations that may be co-applied with OLIF. We predicted and compared the ranges of motion (ROMs) of individual segments, the maximum Mises stresses on fixation instruments (MMSFIs), and the average Mises stresses on cancellous bone (AMSCBs). The effects of osteoporosis on the biomechanics of the various supplemental fixations were analyzed.

## Methods

### 2.1 Model of the normal lumbar spine

The FE model used in this study was part (L3-S1) of a previously established and validated spine model [16, 17, 22]. In brief, modeling proceeded as follows. A complete geometrical model of the lumbar spine was constructed in Mimics 10.0 (Materialise Technologies, Leuven, Belgium) using computed tomography images of a 30-year-old healthy man without any abnormalities. Reconstruction employed Geomagic Studio 10.0 reverse engineering software (Geomagic Inc., NC, USA). HyperMesh 11.0 preprocessing software (Altair Engineering Corp., MI, USA) was used for meshing and assignment of material properties to the vertebral body. Then, FE analysis was performed with the aid of Abaqus 6.11 (Dassault Systems Corp., PA, USA). The complete FE model featured cancellous, cortical, endplate, intervertebral disc, and posterior elements. The cancellous vertebral body was surrounded by a 0.5-mm layer of cortical bone [22, 23]. The intervertebral disc featured a nucleus pulposus and an annulus fibrosus in proportions of 44% and 56% [24], respectively, reflecting the histological composition. The average space between facets was 0.1 mm. The seven ligaments connecting the vertebrae were modeled as tension-only three-dimensional springs. The detailed material properties of each component of the complete model were derived from the literature.

### 2.2 OLIF surgical models

**Fig. 1:**
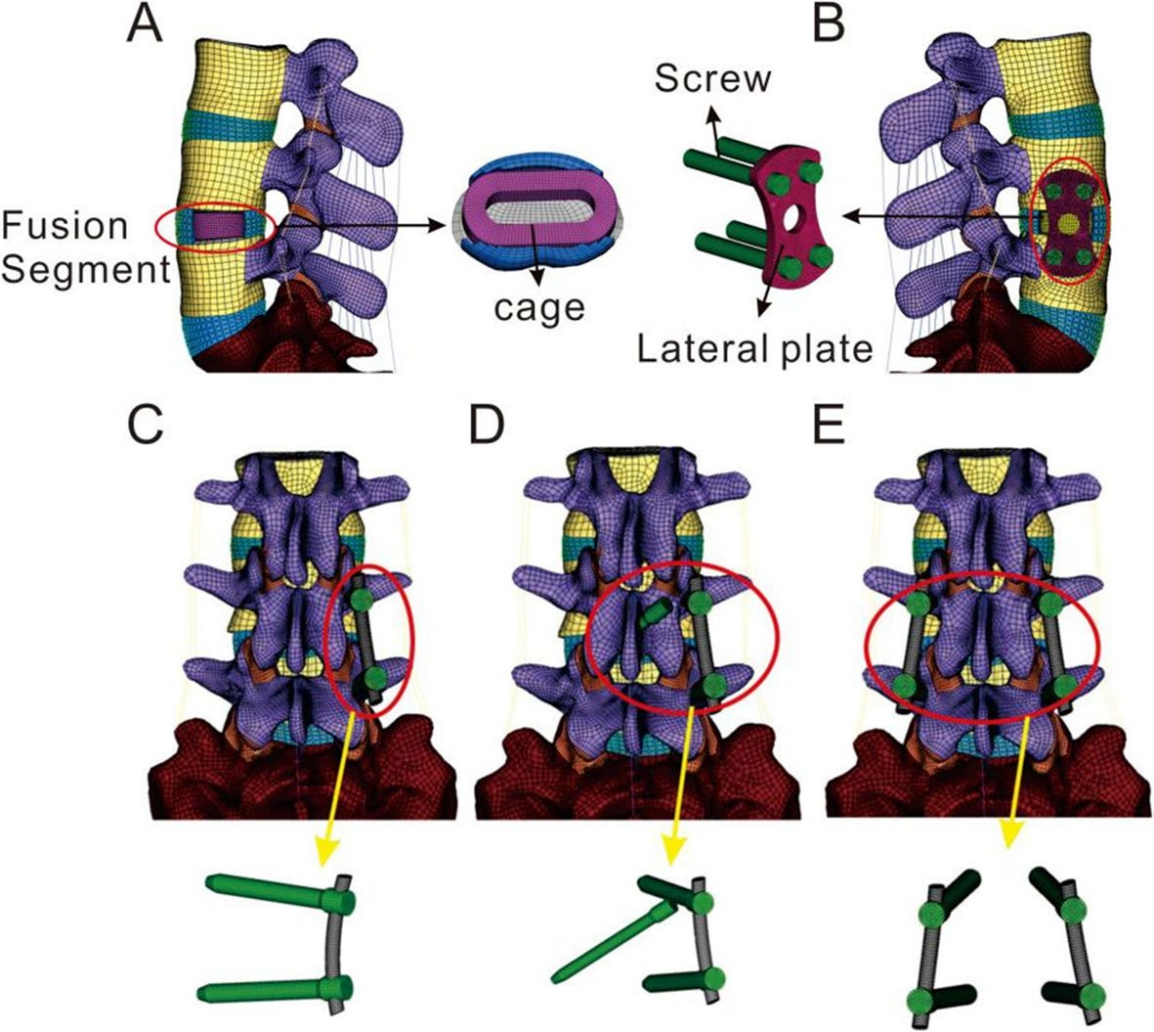
Five OLIF lumbar spine FE models**. A:** Standalone OLIF; **B:** OLIF + LPF; OLIF with lateral plate fixation; **C:** OLIF + UPSF; OLIF with unilateral pedicle and rod fixation; **D:** OLIF + TFJF + UPSF; OLIF with translaminar facet joint fixation and unilateral pedicle and rod fixation; **E:** OLIF + BPSF; OLIF with bilateral pedicle and screw and rod fixation

**Fig. 2:**
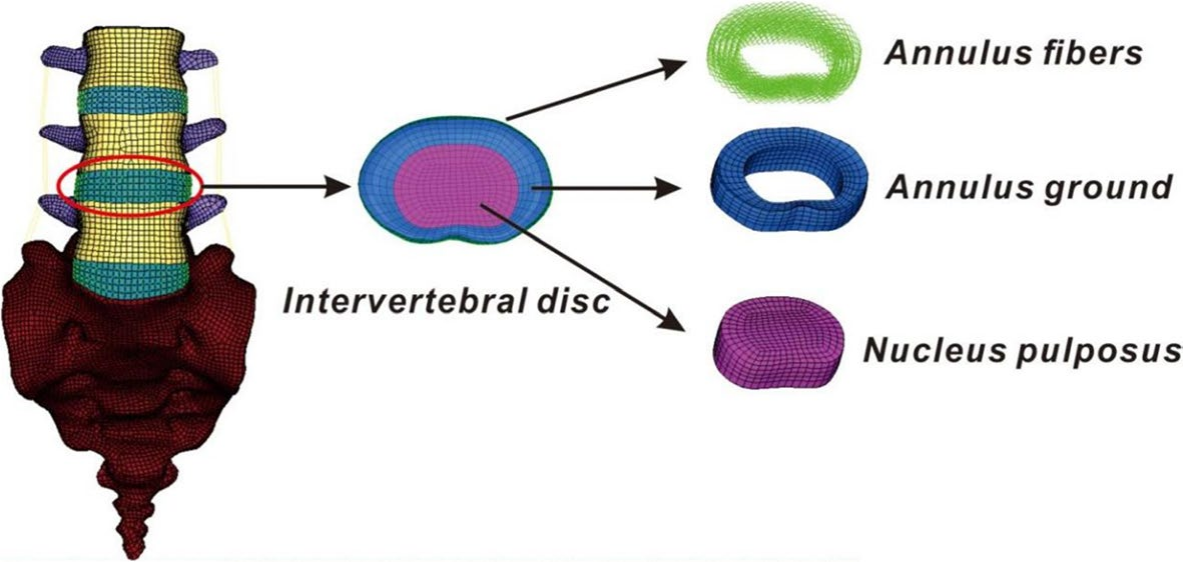
Three-dimensional, nonlinear finite element model of the lumbar spine (L3-S1)

Five supplemental fixations established for OLIF surgical model previously [25], was evaluated on osteoporotic lumbar spines: standalone OLIF; OLIF with lateral plate fixation (OLIF + LPF); OLIF with translaminar facet joint fixation and unilateral pedicle screw fixation (OLIF + TFJF + UPSF); OLIF with unilateral pedicle screw fixation (OLIF + UPSF); and OLIF with bilateral pedicle screw fixation (OLIF + BPSF). We simulated surgery on the L4-L5 segment (Fig. 1) because this is the segment most often affected in asymptomatic individuals [14, 26, 27]. During the simulations, the entire nucleus pulposus and part of the annulus fibrosus were removed (Fig. 2). We then created pedicle screws (diameter, 6.5 mm; length, 45 mm), a rod (diameter, 6.0 mm), and a cage (length, 45 mm; width, 22 mm; average width, 9.5 mm; surface area, 28.21 cm^2^) in SolidWorks. We introduced the cage to the intervertebral discs of the L4-L5 segment, imported IGES files of the screws and vertebrae into HyperMesh, generated holes via Boolean operations, and added different fixation devices to the L4-L5 segments (Fig. 2). Notably, because the rigid fixation system was simulated, the screw-rod and screw-side plate interacted through a common node, while the screw-vertebrae and cage-endplate interacted through a “Tie” constraint. The types of element and material parameters of each component of the normal model are shown in Table 1 [14, 16, 17].

**Table 1:** Normal bone: material properties and elements of the lumbar spine model and the implants

Component	Young’s modulus (MPa)	Poisson’s ratio	Element types
Cortical	12,000 (7920)	0.3	C3D8R
Cancellous	100 (33)	0.3	C3D4
Posterior element	3500 (2310)	0.3	C3D4
Endplate	24 (16)	0.4	C3D8R
Sacrum	5000	0.2	C3D4
Cage	3600	0.25	C3D8R
Screws and rods	110,000	0.3	C3D8R
Lateral plate	110,000	0.3	C3D8R
Facet cartilage	Neo-Hookean, C10 = 2		C3D8RH
Annulus ground	Mooney–Rivlin, C1 = 0.18, C2 = 0.045		C3D8RH
Nucleus pulposus	Mooney–Rivlin, C1 = 0.12, C2 = 0.03		C3D8RH
Annulus fibers	Calibrated stress–strain curves		Spring
Seven ligaments	Calibrated deflection–force curves		Spring

Seven ligaments: anterior longitudinal ligament; posterior longitudinal ligament; intertransverse ligament; ligamentum flavum; supraspinous ligament; interspinous ligament; capsular ligament

### 2.3 Osteoporotic vertebral material properties

According to a previous study [28], osteoporosis changes the elastic moduli of all bones in the vertebral body without significant changes in other material parameters. The thickness of lumbar spine cortical bone does not significantly change. Thus, when creating the osteoporotic FE model, the modulus of elasticity was reduced by 33% for cortical bone and the endplate and posterior structures; it was reduced by 66% for all cancellous bone structures [28–30]. All other material parameters were unchanged. The vertebral and posterior structures were considered to be isotropic and homogeneously elastic. The material properties and elements of the osteoporotic components are listed in Table 2.

**Table 2:** Osteoporotic bone: material properties and elements of the lumbar spine model

Component	Young’s modulus (MPa)	Poisson’s ratio	Element types
Cortical	7920 (↓ 33%)	0.3	C3D8R
Cancellous	33 (↓ 66%)	0.3	C3D4
Posterior element	2310 (↓ 33%)	0.3	C3D4
Endplate	16 (↓ 33%)	0.4	C3D8R

### 2.4 Boundary and load conditions

**Fig. 3:**
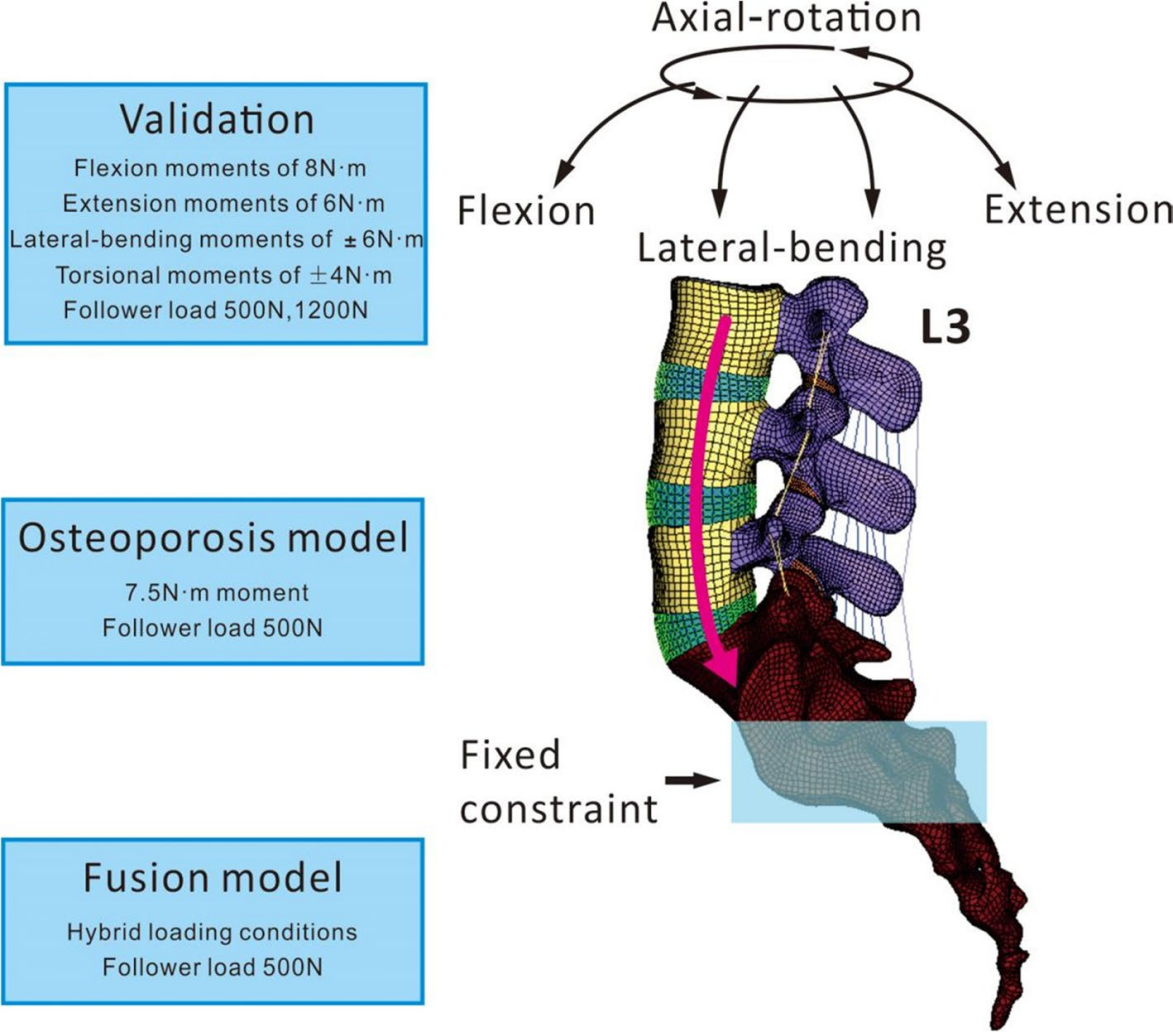
Schematic of torque and follower loads applied to the lumbar spine (L3-S1)

Coupling points were set in the centers of the upper and lower endplate surfaces of each vertebral body; seven such points were set in the L3-S1 segment. Connector elements were created through the coupling points. As shown in Fig. 3, the follower load is a physiological compressive load along the axis of the spine. A follower load of 500 N was applied to each level of the lumbar spine through the above mentioned connector elements. The follower loads were applied as suggested in the literature [17, 31]. Under a follower load of 500 N, a moment load of 7.5 N·m was applied to the upper surface of the upper endplate of L3 to simulate six different postures: flexion, extension, left bending, right bending, left axial rotation, and right axial rotation. During the entire loading process, the translational and rotational degrees of freedom of S1 were limited in the X, Y, and Z axes [32].

### 2.5 Mesh sensitivity check

**Fig. 4:**
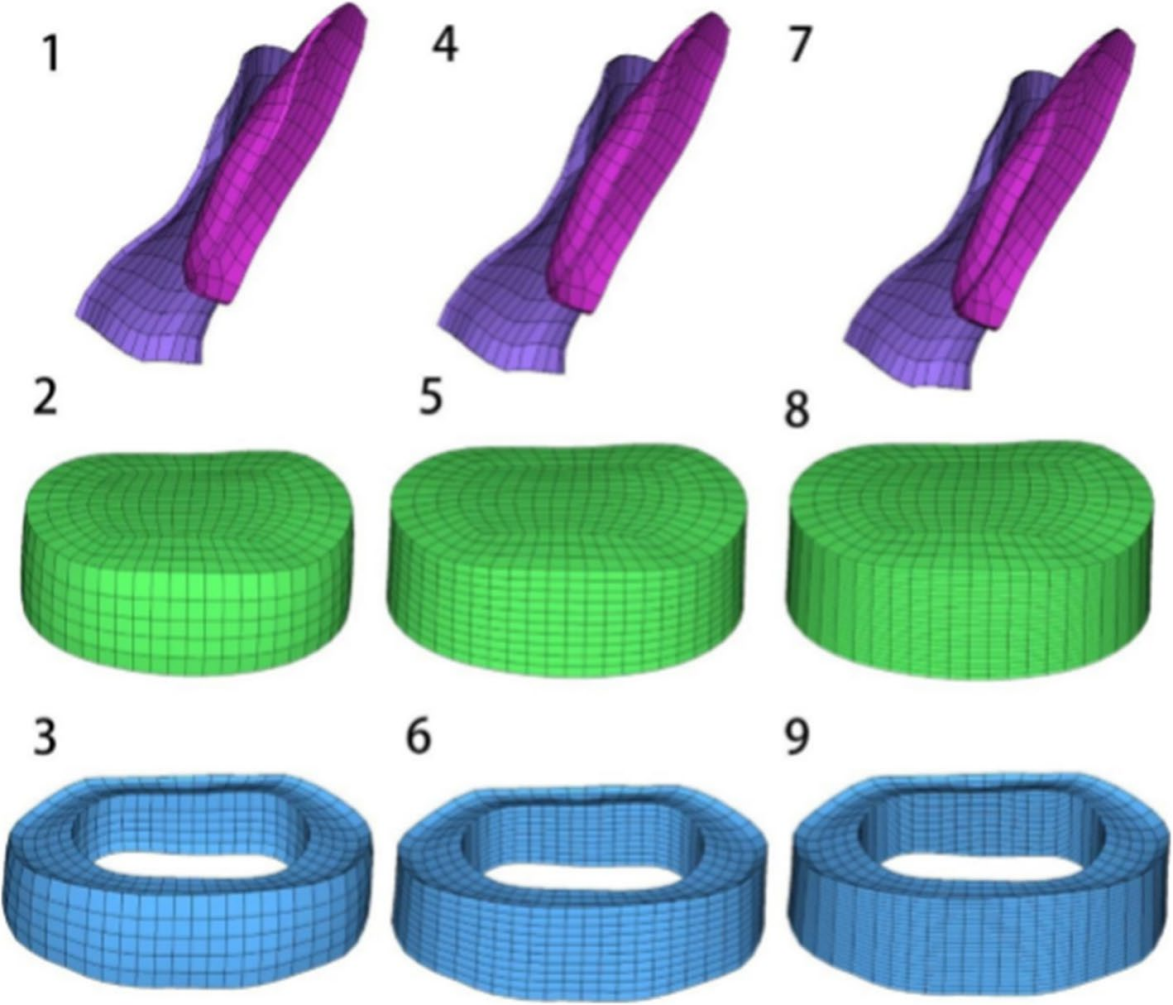
Schematic mesh refinement of annulus ground, nucleus pulposus and facet cartilage in the normal model. (1–3 = No refinement, 4–6 = Mesh refinement-2X, 7–9 Mesh refinement-4X)

In this paper, the mesh sensitivity of the developed lumbar spine model was examined. Since each spinal structure affects the forces on the lumbar spine differently, the two main parts of the lumbar spine that are subjected to forces were selected: the small joints and the discs. In addition, since each spinal motion unit of the lumbar spine has the same effect, the L4-L5 segments were randomly selected. As shown in the Fig. 4, the meshes of the small articular cartilage, nucleus pulposus and fibrous annulus matrix were refined by a factor of 2X and 4X, respectively, and then the corresponding biomechanical parameters were calculated and compared with the calibrated and validated normal model of the lumbar spine described above. As shown in Table 3, In posterior extension and left rotation positions, the corresponding biomechanical parameters (range of motion, intradiscal pressure and cartilage forces) did not vary by more than 1%. Therefore, it can be concluded that this normal lumbar spine model is convergent and can indicate that the current mesh refinement is appropriate.

**Table 3:** Mesh refinement of the annulus ground, nucleus pulposus and facet cartilage in the L4-L5 segment of the normal lumbar spine model. (*Ext* Extension, *LAR* Left rotation, *L* Left, *R* = Right)

Mesh refinement	Annulus ground (number)	Nucleus pulposus (number)	Facet cartilage (number)	ROM (°)	cartilage force (N)	IDP (MPa)
Normal (No refinement)	1200	1400	338	Ext = 5.53 LAR = 2.55	Ext(L) = 108.16 Ext(R) = 101.95 LAR(R) = 126.83	Ext = 0.678 LAR = 0.531
Mesh refinement (2X)	2400	2880	676	Ext = 5.54 LAR = 2.58	Ext(L) = 108.19 Ext(R) = 101.98 LAR(R) = 126.72	Ext = 0.674 RAR = 0.538
Mesh refinement (4X)	4800	5760	1352	Ext = 5.52 LAR = 2.54	Ext(L) = 108.21 Ext(R) = 101.88 LAR(R) = 126.62	Ext = 0.675 LAR = 0.534

## Results

### 3.1 ROMs

**Fig. 5:**
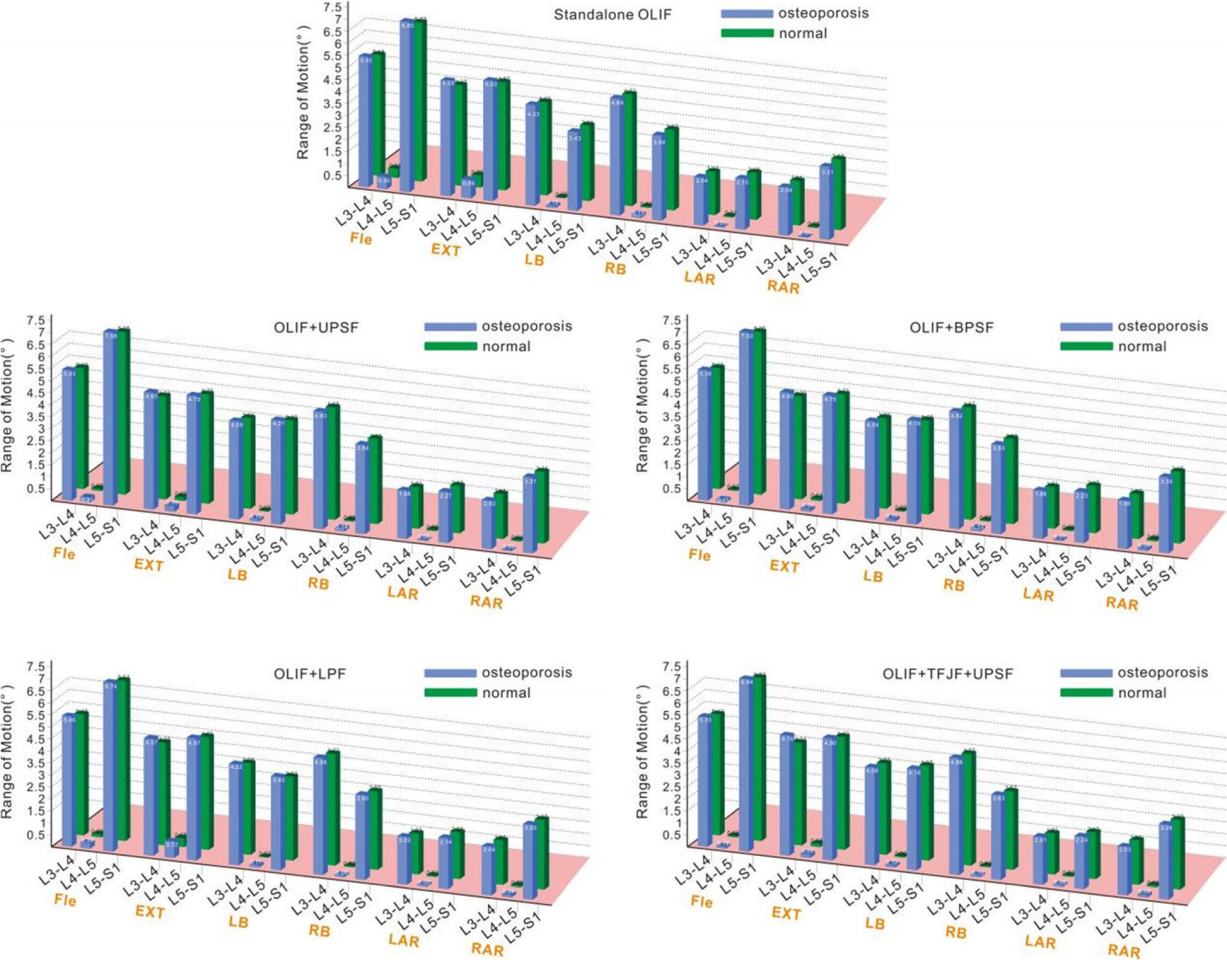
ROMs of various segments of osteoporotic and normal bones

Figure 5 shows the ROMs of the L3-S1 segments of different models (five OLIF models each for normal and osteoporotic bone) in the six postures. OLIF for patients with osteoporotic or normal bone significantly reduced the ROM of the L4-L5 surgical segment. The overall segmental ROM of the normal model decreased, compared with the overall segmental ROM of the osteoporotic model, principally because the ROM of the operative segment decreased. When the various supplemental fixations were applied, the osteoporotic OLIF models revealed no obvious change in the ROM of any segment, compared with the ROM of the normal bone OLIF model.

### 3.2 MMSFIs under normal and osteoporotic conditions

**Fig. 6:**
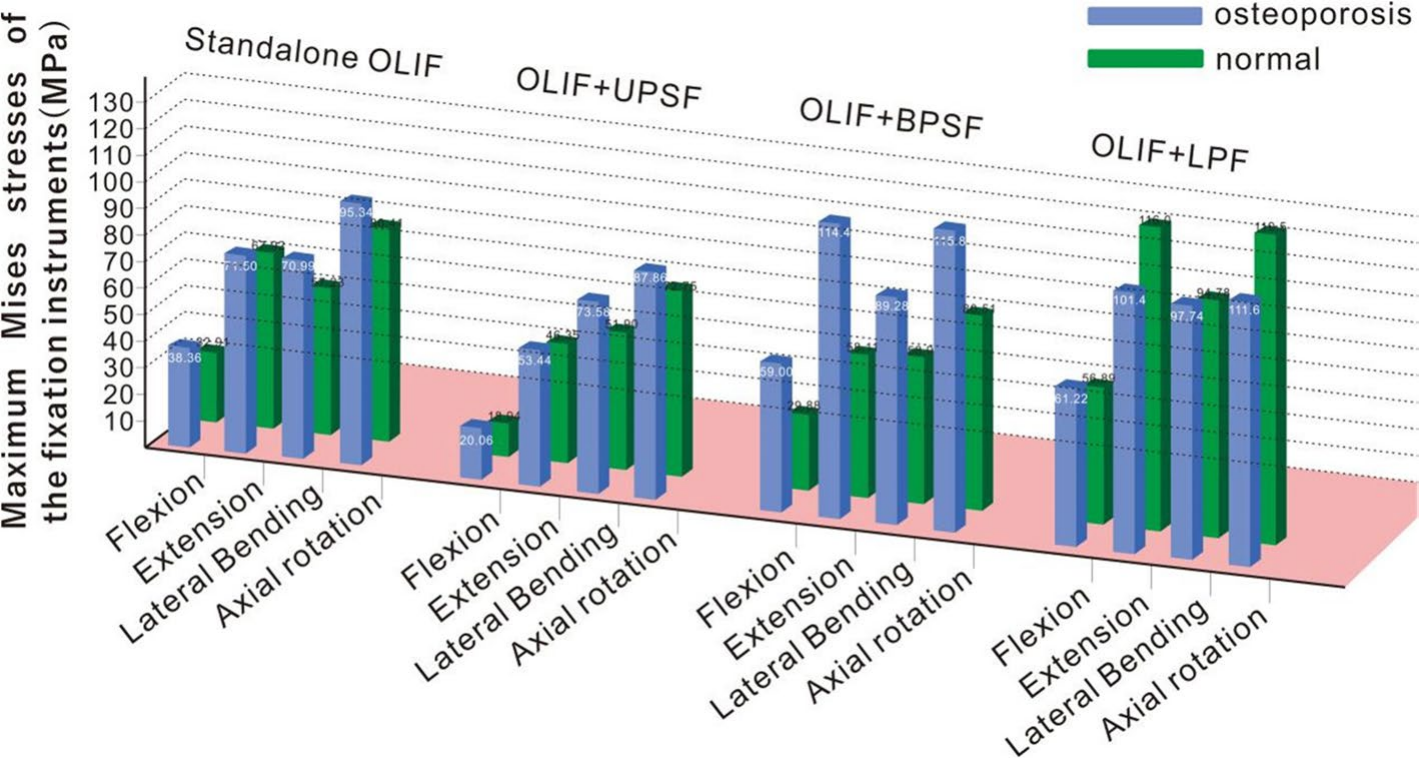
Maximum Mises stresses of the fixation instruments (MMSFIs) of normal and osteoporotic bones

**Fig. 7:**
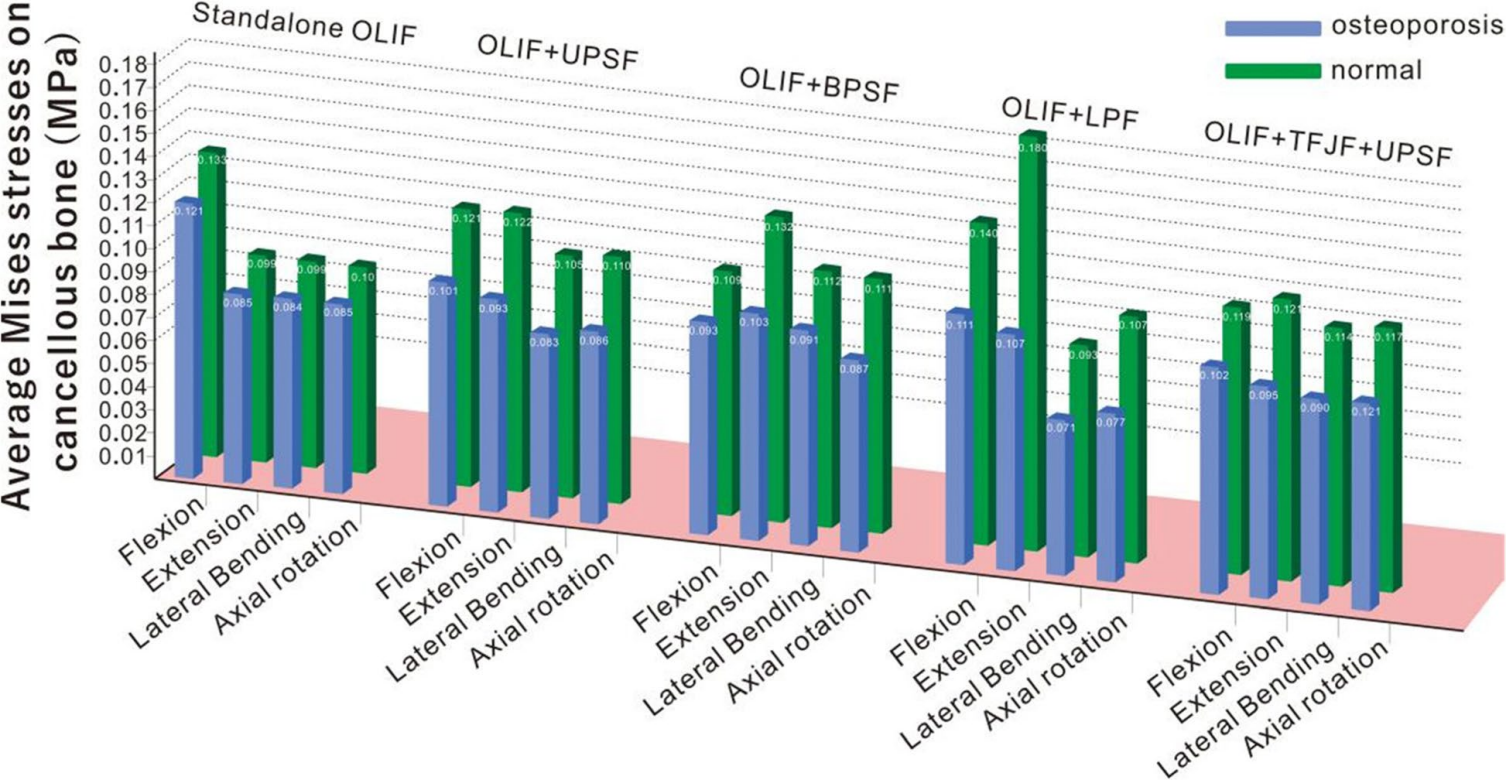
Average Mises stresses (AMSCBs) of cancellous normal and osteoporotic bones

The peak Mises stresses on the various supplemental fixation instruments (in different postures) of both models are shown in Fig. 6. Notably, MMSFIs were detected in both the osteoporotic and normal bone models under various working conditions. The MMSFIs in osteoporotic bone were larger than the MMSFIs in the normal bone surgical model. Under flexion, MMSFIs were detected on the supplemental fixation instruments of both the osteoporotic and normal bone surgery models (Fig. 7). The MMSFIs of the OLIF + UPSF and OLIF + BPSF models increased by 16.56% (from 32.91 to 38.36) and 16.47% (from 18.94 to 22.06 MPa), respectively. In the OLIF + TFJF + UPSF model, the MMSFI increased sharply from 29.88 to 59.00 MPa; in the OLIF + LPF model, the MMSFI changed slightly from 56.89 to 61.22 MPa (a 7.61% increase). Similarly, the stresses on the supplemental fixation instruments of the osteoporotic model were highest after extension, as in the normal bone model (Fig. 6). In the OLIF + TFJF + UPSF model, the MMSFI increased sharply with decreasing bone mass (from 59.11 to 114.40 MPa). In the OLIF + LPF model, the MMSFI decreased from 101.40 to 116.01 MPa (a reduction of 14.41%). However, in the OLIF + UPSF and OLIF + BPSF models, the MMSFIs were similar to the values for flexion. Compared with the normal bone model, the osteoporotic model imposed higher stresses on the supplemental fixation instruments.

The maximum stresses on the supplemental fixation instruments during lateral bending and axial rotation are shown in Fig. 6. Both the osteoporotic and normal models exhibited MMSFIs. Comparisons revealed that the stress changes of the OLIF + UPSF, OLIF + BPSF, and OLIF + TFJF + UPSF models during lateral bending were similar to the values on flexion and extension; all stresses trended upward. The changes were most obvious in the OLIF + TFJF + UPSF model. However, in the OLIF + LPF model (compared with the normal bone surgery model), the stresses on the supplemental fixation instruments increased by 3.12% (from 94.78 to 97.74 MPa) and 1.37% (from 114.62 to 116.19 MPa), respectively.

### 3.3 AMSCBs

As shown in Fig. 7, under different loads, the AMSCBs of the five OLIF osteoporotic models decreased, compared with the values of the normal bone model. AMSCBs were evaluated because it is important to eliminate stress concentrations caused by perforations of cancellous bone. In the osteoporotic model, the AMSCBs were markedly altered (Fig. 7). Under flexion, the comparison indicated that the AMSCB decreased from 0.133 to 0.121 MPa in the standalone OLIF model; it decreased from 0.121 to 0.101 MPa in the OLIF + UPSF model, 0.109 to 0.093 MPa in the OLIF + BPSF model, 0.14 to 0.111 MPa in the OLIF + LPF model, and 0.119 to 0.102 MPa in the OLIF + TFJF + UPSF model. Notably, the average stress changes on cancellous bone were most obvious under extension. The AMSCBs of the five OLIF models decreased by 14%, 23.44%, 21.97%, 40.56%, and 22.44%, respectively. Of these, the OLIF + LPF change was most obvious: from 0.18 to 0.107 MPa. Importantly, during lateral bending and axial rotation, the AMSCBs were similar to the values under flexion and extension. Compared with the values of the normal bone model, the AMSCBs decreased to varying extents.

### 3.4 Maximum stress of cage

**Fig. 8:**
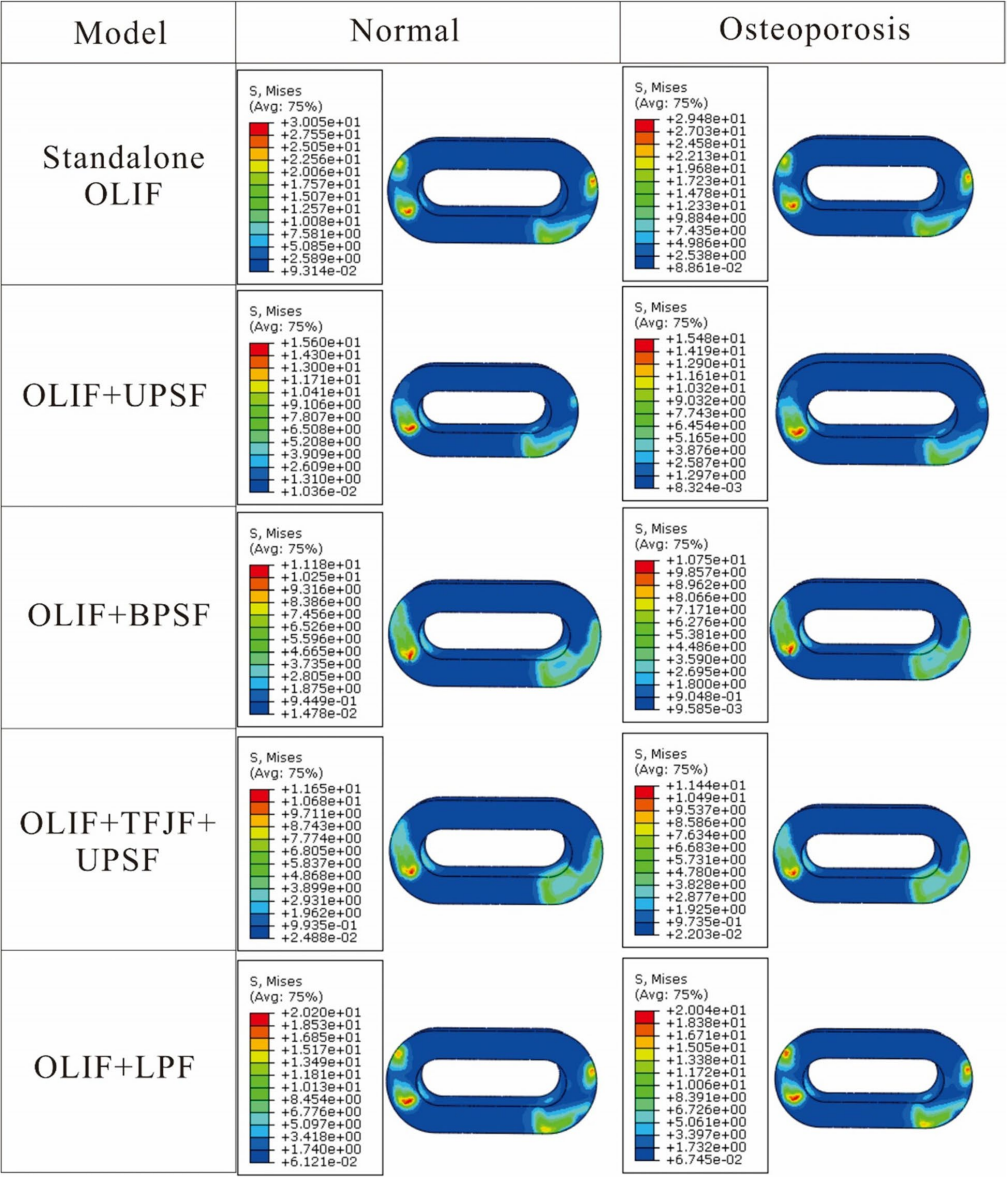
Von Mises stress distribution on the cage, with various supplemental fixations co-applied

The von Mises stress distribution on the cage was randomly selected to be shown by contour plots in the forward flexion posture (Fig. 8). The results showed that the stress distribution was similar for osteoporotic and normal bone cages under the same fixation method. However, the stresses on the cages of patients with osteoporosis were all slightly higher than those of normal bone. Also, the smallest mises stresses were found in OLIF + UPSF and OLIF + BPSF.

## Discussion

OLIF is favored by spinal surgeons because it is associated with less bleeding, a shorter operative time, and faster recovery, compared with other lumbar disc fusion surgeries [33, 34]. Although OLIF can achieve excellent results and is widely used in practice, it can destabilize the spine and increase the risks of cage subsidence and fracture [8, 35, 36]. In patients with osteoporosis, these risks may be greater because of the reduced bone mass and increased degradation of bone, compared with those characteristics in patients with normal bone mass; osteoporotic bone is more brittle. In earlier reports, patients with osteoporosis were prone to complications such as screw loosening and extraction, which increased internal fixation failure [37–39]. However, the effects of OLIF co-applied with various supplemental fixations in osteoporotic patients remain unknown. Therefore, we built FE models of osteoporotic and normal spines, then applied various supplemental fixations to determine their biomechanical responses.

To explore the effects of osteoporosis on various supplemental fixations that may be co-applied with OLIF, we established and verified a three-dimensional, nonlinear complete FE (L3-S1) model. Then, we endowed each part of the lumbar spine with osteoporotic features to establish a three-dimensional, nonlinear, osteoporotic FE model. Appropriate modifications were made at the L4-L5 level; we simulated five OLIF models using different fixed instruments.

After a patient has undergone OLIF surgery, the stability of the surgical segment is an important index of rehabilitation that greatly concerns clinicians [40] because instability is usually associated with various complications such as intervertebral interstitial inflammation, cage subsidence, reduced intervertebral disc height, and vertebral body non-fusion. Lu et al. [4] used an FE model to explore the biomechanical properties of four LIF surgeries (PLIF, TLIF, XLIF, and OLIF). The ROMs of the surgical segments were reduced. Chen et al. [41] established single-segment lateral interbody fusion surgical models; they found that, compared with the complete model, the ROMs decreased by 76.84–97.97%. Oxland et al. [42] reviewed the biomechanical characteristics of LIF surgeries. The maximum ROM reduction at the index level was 90%. We found that OLIF co-applied with various supplemental fixations in osteoporotic patients significantly reduced the ROM of the L4-L5 surgical segment; thus, it afforded good surgical site stability. As shown in Fig. 6, the ROMs of the five supplemental fixation instruments in different positions did not differ markedly from the ROMs of normal bone, either for the surgical segment (L4-L5) or the non-fused segment. These results indicated that osteoporosis did not greatly affect the ROM of the lumbar spine, consistent with the findings in previous studies [43]. Therefore, OLIF surgery using the same fixation modality in osteoporotic patients does not affect the vertebral body ROM to a greater extent than in patients with normal bone quality. Previous studies found that a reduced spinal ROM was usually associated with disc degeneration [44, 45]. Because the intervertebral discs of the osteoporotic spine are not markedly degenerative, it is unsurprising to find that their mobility is not significantly affected.

In the osteoporotic model, the stresses on the vertebrae and supplemental fixations changed greatly, compared with stresses in the normal model). Under axial compression, the displacement of osteoporotic vertebrae is greater than the displacement of normal vertebrae; however, considering the lower strength of osteoporotic vertebrae, as well as the small displacements of the screw and rod, the load is transferred to the screw and rod. Under flexion, osteoporotic vertebrae are less stiff than normal vertebrae and exhibit deformation; screw displacement is reduced and more torque is generated between the vertebrae and the screw, imparting high-level stress to the screw. Moreover, the increased vertebral displacement enhances screw and rod displacement; the rods bend more and thus experience higher stresses. Similarly, under extension, the osteoporotic vertebral body is softer and more deformed than the normal vertebral body; a small screw displacement may increase the distance between the vertebral body and the screw, concentrating stress on the screw. Considering the softer osteoporotic vertebral body, compared with normal bone, greater stresses are imparted to the supplemental fixation instruments; this is consistent with the above findings for the rod.

Notably, during standalone OLIF, only the average stress on the vertebrae was analyzed; no supplemental fixation instrument was placed. Compared with normal lumbar spine surgery, standalone OLIF produced less average stress, similar to the results in other fixation systems. This is presumably because osteoporotic vertebrae are softer than normal vertebrae; thus, they impart less stress. The stress levels on the vertebrae and the supplemental fixations in the osteoporotic model are reduced and increased, respectively, in the various postures, compared with those values in the normal bone model; these findings are consistent with the results of previous studies [46]. Therefore, the biomechanical properties of the osteoporotic model are less robust than the properties of the normal model when the same supplemental fixation method is employed.

We found that osteoporosis affected OLIF to various extents, depending on the chosen supplemental fixation system. Fixation affords strong support and can prevent fractures. In the osteoporotic model, stresses on the proximal-junction vertebrae are reduced. However, the stress on a proximal fixation system increases, stress becomes concentrated on the contact interface between the cone and the screw, which is associated with an increased risk of internal fixation failure. Osteoporosis reduces the vertebral elastic modulus and tensile strength. Such changes may increase the relative displacement between the vertebrae and the fixation system in the same radial direction; this may cause the internal fixation device to loosen or rupture. A high pressure at the contact interface between the vertebrae and the screw can trigger bone destruction, such as a fracture. Also, due to the low Young's modulus of osteoporotic vertebrae, the difference in mechanical properties between bone and cage leads to increased bone stress and decreased cage stress, indirectly leading to an increased risk of cage subsidence. Therefore, the fixation system chosen and the vertebral strength should be considered when performing OLIF in osteoporotic patients. Firm fixation and a strong vertebral body are essential for the long-term maintenance of fractures repaired after exposure to high stress; they are also essential for reducing the risk of internal fixation failure.

There are several limitations should be addressed. First, our FE model of the lumbar spine was based on geometrical information from one person. The osteoporotic FE model was constructed by ignoring individual differences and reducing the elastic moduli of the endplate, as well as the cortical, cancellous, and posterior elements, by specific proportions. Second, although FE analysis affords many advantages for assessment of biomechanics, compared with in vitro experiments, the inability to reconstruct muscles is a common problem experienced by all current lumbar FE models. Furthermore, FE methods do not closely simulate the true geometry of ligaments; these are simplified to one-dimensional non-linear springs. Finally, the results of FE analysis reflect only the post-surgical condition, rather than the long-term postoperative status. Despite these limitations, the response parameters of our lumbar spine FE model are consistent with published in vitro experimental data concerning spinal biomechanics. Therefore, clinicians may find our results useful.

## Conclusions

This study simulated OLIF surgery in patients with osteoporosis and degenerative disease. We evaluated the utilities of various supplemental fixation methods. Bilateral pedicle screw fixation (BPSF) provided superior primary biomechanical stability for OLIF cages, compared to Standalone, lateral plate fixation (LPF), translaminar facet joint fixation and unilateral pedicle screw fixation (TFJF + UPSF), unilateral pedicle screw fixation (UPSF) under different bone conditions. Osteoporosis amplifies the difference in stability between BPSF and the other four fixation methods. Clinically, in conditions of decreased bone quality, the surgeon must consider the limitations of Standalone, LPF, TFJF + UPSF, and UPSF fixation, although the first two have the advantage of not requiring secondary surgery with percutaneous pedicle screw fixation and the latter two have the advantage of being less destructive to the bone. This study highlights the need for further investigations (experimental and clinical trials) on the improvement of supplemental instruments and to adapt fixation methods in OLIF to the patient's bone quality to reduce the risk of internal fixation failure.

## Data Availability

The datasets used and/or analyzed during the current study are available from the corresponding author on reasonable request. **Ethics approval and consent to participate** Our study was approved by the institutional ethics committee of the Tianjin Hospital. All procedures were performed in accordance with relevant guidelines.Written informed consent was obtained from all participants included in this study. **Consent for publication** Not Applicable.

## Abbreviations

OLIF: Oblique lumbar interbody fusion
FE: Finite element
OLIF + LPF: OLIF with lateral plate fixation
OLIF + TFJF + UPSF: OLIF with translaminar facet joint fixation and unilateral pedicle screw fixation
OLIF + UPSF: OLIF with unilateral pedicle screw fixation
OLIF + BPSF: OLIF with bilateral pedicle screw fixation
Rom: Ranges of motion
MMSFIs: The maximum Mises stresses of the fixation instruments
AMSCBs: The average Mises stresses on cancellous bone
